# A Novel Singular Value Decomposition-Based Denoising Method in 4-Dimensional Computed Tomography of the Brain in Stroke Patients with Statistical Evaluation

**DOI:** 10.3390/s20113063

**Published:** 2020-05-28

**Authors:** WonSeok Yang, Jun-Yong Hong, Jeong-Youn Kim, Seung-ho Paik, Seung Hyun Lee, Ji-Su Park, Gihyoun Lee, Beop Min Kim, Young-Jin Jung

**Affiliations:** 1 Department of Radiology, Dong-A University Hospital, Busan 49201, Korea; ywsuck@naver.com; 2 Department of Multidisciplinary Radiological Science, Graduate School, Dongseo University, Busan 47011, Korea; hohohong6161@hanmail.net; 3 Clinical Emotion and Cognition Research Laboratory, Inje University Ilsan Paik Hospital, Goyang 10380, Korea; jeongyounk@naver.com; 4 Department of Bioengineering, Korea University, Seoul 02841, Korea; paikjang@korea.ac.kr (S.-h.P.); aksska82@korea.ac.kr (S.H.L.); 5 KLIEN Inc., Seoul Biohub, 117-3, Hoegi-ro, Dongdaemun-gu, Seoul 02455, Korea; 6 Advanced Human Resource Development Project Group for Health Care in Aging Friendly Industry, Dongseo University, Busan 47011, Korea; jisu627@hanmail.net; 7 Department of Physical and Rehabilitation Medicine, Center for Prevention and Rehabilitation, Heart Vascular Stroke Institute, Samsung Medical Center, Sungkyunkwan University School of Medicine, Seoul 02841, Korea; gihyounlee@gmail.com

**Keywords:** acute stroke, computed tomography, image quality, singular value decomposition, Gaussian noise, contrast-to-noise, signal-to-noise ratio, brain, radiation doses

## Abstract

Computed tomography (CT) is a widely used medical imaging modality for diagnosing various diseases. Among CT techniques, 4-dimensional CT perfusion (4D-CTP) of the brain is established in most centers for diagnosing strokes and is considered the gold standard for hyperacute stroke diagnosis. However, because the detrimental effects of high radiation doses from 4D-CTP may cause serious health risks in stroke survivors, our research team aimed to introduce a novel image-processing technique. Our singular value decomposition (SVD)-based image-processing technique can improve image quality, first, by separating several image components using SVD and, second, by reconstructing signal component images to remove noise, thereby improving image quality. For the demonstration in this study, 20 4D-CTP dynamic images of suspected acute stroke patients were collected. Both the images that were and were not processed via the proposed method were compared. Each acquired image was objectively evaluated using contrast-to-noise and signal-to-noise ratios. The scores of the parameters assessed for the qualitative evaluation of image quality improved to an excellent rating (*p* < 0.05). Therefore, our SVD-based image-denoising technique improved the diagnostic value of images by improving their quality. The denoising technique and statistical evaluation can be utilized in various clinical applications to provide advanced medical services.

## 1. Introduction

Strokes have become a major issue affecting aging populations worldwide [1,2,3], which makes appropriate prevention and treatment measures crucial. It is very important to seek emergency care during the initial manifestations of the signs and symptoms of stroke; when ischemic or hemorrhagic strokes occur in the brain, the blood supply to the brain stops causing brain cells to die quickly, which could be fatal for patients [4]. According to a study conducted by the National Institute of Neurological Disorders and Stroke (NINDS) that is part of the National Institutes of Health (NIH), an intravenous injection of recombinant tissue plasminogen activator (r-TPA) administered within 3 h of the onset of symptoms is significantly effective in treating stroke patients without them having to undergo surgery [5,6].

Nowadays, to identify the affected sites that could lead to strokes, computed tomography perfusion (CTP) and magnetic resonance imaging (MRI) scans are commonly performed for clinical diagnoses [7,8,9]. Both of these imaging modalities have different strengths and weaknesses. Clinically, although MRI is the front-runner among imaging modalities for the detection of affected sites in stroke patients involving no risk of them being exposed to radiation [10], the long examination time that may be required limits its practical clinical use [11]. For this reason, CTP has been used in the diagnosis of acute stroke and has reduced the imaging examination time.

However, 4-dimensional (4D) CTP is generally considered to result in the patient being exposed to a higher radiation dose owing to the associated need for repeated sequential 3-dimensional (3D) CT scans over time. In 2009, the Food and Drug Administration (FDA) reported that patients who underwent a CT scan of the brain exhibited band-like hair loss and, therefore, issued a warning regarding the excessive radiation dose that patients are subjected to [12]. Furthermore, CTP is a relatively high-dose technique because of the patients’ repetitive exposure to radiation throughout the scanning duration. In order to reduce the radiation dose that patients are exposed to, a low-dose scanning technique has been investigated [13]; however, it was found that it generates Gaussian noise on the diagnostic images, which degrades their quality.

As the quality of the image deteriorates, the diagnostic success rates associated with using these images may also decrease. The Gaussian noise that is generated by scanning the brain at low radiation doses interferes with the accurate interpretation of these images and with the identification of brain-blood vessels as well as of cerebral parenchyma [14]. Therefore, the use of higher radiation doses appears to be unavoidable in order to improve the quality of images. Due to these issues, original axial CTP images obtained via the conventional method are only used to record hemodynamic parameters, such as cerebral blood volume (CBV), cerebral blood flow (CBF), and mean transit time (MTT), rather than being directly used in clinical diagnoses [15].

Owing to the current situation that is outlined above, a need arises to improve axial CTP image quality in order to facilitate the direct use of CTP images in clinical diagnosis. However, previous studies have only focused on controlling radiation exposure by techniques, such as modifying the tube voltage, tube current, and tube rotation time [16,17]. Therefore, in this study, we aim to introduce a singular value decomposition (SVD)-based image analysis technique that improves the axial image quality of CTP images with no additional radiation exposure by focusing on the removal of noise components on axial CTP images.

Based on our results demonstrating improvements in quantitative image quality, our main conclusions were that the SVD-based denoising technique for 4D CTP images has the potential to improve the quality of images captured using lower tube current settings and does not require an increase in the radiation dose.

## 2. Material and Methods

### 2.1. Patient Groups

An institutional review board (IRB) approval was obtained for the use of clinical CTP data in this study before it was carried out (committee’s reference number: KUGH 2016-11-019). The CTP image data of cases involving 20 stroke-suspected patients were collected retrospectively. The data were acquired from 1 January 2016 to 1 December 2016 at Kosin University Medical Center. Patients who were already diagnosed with ischemic or hemorrhagic strokes on CT images were excluded.

### 2.2. Data Acquisition and Reconstruction

CTP examinations were performed using a 128-section dual-source CT scanner (SOMATOM Definition-Flash, Siemens Healthcare, Forchheim, Germany). Following a CT scan of the whole brain without the use of a contrast agent, CTP scans of 84 mm scanned slices of the cranial area including the basal ganglia were performed at 80 kVp and 200 mAs with a 0.28 s rotation time. Each scan was acquired with a detector collimation of 32 × 1.2 mm and a temporal interval of 1.25 s; these settings were applied via a volume shuttle spiral scanning mode called AD4S. Regarding the contrast injection protocol, a 40 cc volume of a non-ionic contrast media (Scanlux, 370 mgl/mL) was administered at a rate of 6 mL/s via an intravenous cannula into the antecubital vein using an automatic injector (Stellant D Dual Syringe, MEDRAD Inc., Whippany, NJ, USA), followed by 60 cc of normal saline at the same injection rate as that of the contrast medium. Before the administration of the contrast medium injection, 30 cc of saline was injected to confirm the patency of the intravenous access line. All the patients were examined for 38 s each. Scan delay was set to 2 s, and after scanning, 84 mm of the scanned area was reconstructed at a slice thickness of 5 mm with a 3 mm recon interval. During this process, 28 levels of adjacent images with an overlap of 2 mm were captured.

### 2.3. The SVD Technique

SVD is a mathematical technique that is employed to decompose multidimensional matrix data, such as CTP images [18,19,20]. The SVD technique is commonly used in various clinical research fields for the following purposes: extracting relevant information from heterogeneously mixed multidimensional datasets, reducing the data dimension, and identifying simplified dynamics within noisy data. The SVD equation is generally denoted as follows:

$$A=USV^{\prime }\;=\left[\begin{array}{ccc} u_{1,1} & \cdots & u_{1,m}\\ \vdots & \ddots & \vdots \\ u_{m,1} & \cdots & u_{m,m} \end{array}\right]\left[\begin{array}{ccccccc} \sqrt{\lambda_{1}} & 0 & \ldots & 0 & 0 & \ldots & 0\\ 0 & \sqrt{\lambda_{2}} & \ldots & 0 & 0 & \ldots & 0\\ \vdots & \vdots & \ddots & \vdots & \vdots & \ldots & 0\\ 0 & 0 & \ldots & \sqrt{\lambda_{k}} & 0 & \ldots & 0\\ 0 & 0 & 0 & 0 & 0 & \ldots & 0\\ \vdots & \vdots & \vdots & \vdots & \vdots & \ddots & \vdots \\ 0 & 0 & 0 & 0 & 0 & \ldots & 0 \end{array}\right]\left[\begin{array}{ccc} v_{1,1}^{T} & \cdots & v_{1,n}^{T}\\ \vdots & \ddots & \vdots \\ v_{n,1}^{T} & \cdots & v_{n,n}^{T} \end{array}\right]$$

where *A* is the *m* × *n* matrix, *U* is the *m* × *m* orthogonal matrix comprised of the left singular vector of *A*, and *V* is the *n* × *n* orthogonal matrix comprised of the right singular vector. *S* is the *m* × *n* diagonal matrix comprised singular values, such as $\sqrt{\lambda_{1}}$, $\sqrt{\lambda_{2}}$, …, $\sqrt{\lambda_{k}}$, when the non-zero eigenvalue of *A^T^A* is *λ*_1_, *λ*_2_, …, *λ*_k_.

In recent times, the SVD-based spatio-temporal feature extraction technique has been employed to identify different biomaterials while using various imaging modalities [21,22,23,24]. Previous studies have provided more detailed information on this imaging process [15,25].

### 2.4. Denoising Based on SVD

**Figure 1:**
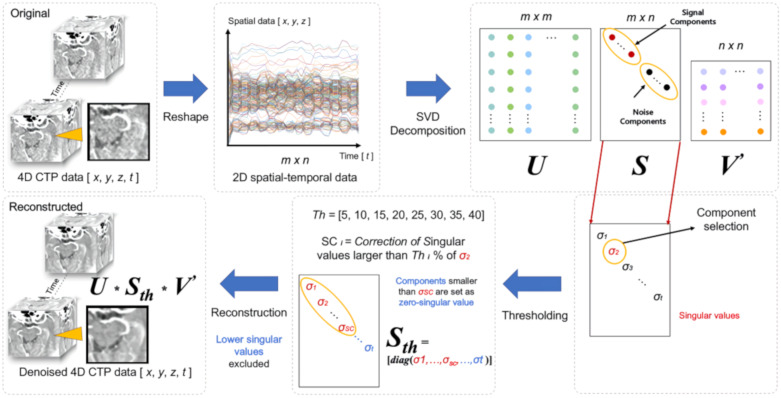
Flow chart of singular value decomposition (SVD)-based denoising technique.

**Figure 2:**
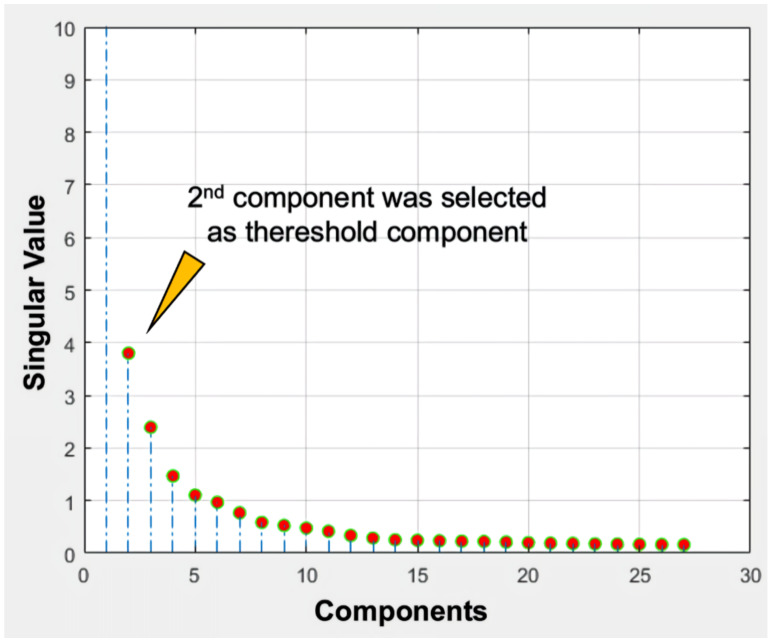
Example of a singular value corresponding to a used dataset. The 2nd component was selected to determine the threshold of the denoised image.

SVD is an internal function of the MATLAB software (MathWorks Inc., Natick, MA, USA,) program [15]. Using SVD, the analyzing software application was developed and used to decompose 4D CTP data in order to exclude noise components on given CTP images (Figure 1) [26]. To use SVD to decompose 4D datasets, the original 4D CTP dataset was reshaped into a 2D spatio-temporal data matrix, and subsequently SVD was performed on the 2D data matrix. The singular values included in the *S* matrix are mainly taken into consideration in order to analyze signal components and noise components. To separate suspected noise components from the original *S* matrix, the following process was performed. First, the *2*nd component, $\sqrt{\lambda_{2}}$, was selected as the thresholding component (threshold value) in accordance with experiential evidence (Figure 2). The threshold value was determined to be 5~40% (increasing interval of 5%) of the $\sqrt{\lambda_{2}}$ singular value. From the diagonal *S* matrix, singular values that were greater than the threshold value were included as the new *S_th_* diagonal matrix, and singular values that were lesser than the threshold value were set as zero-singular values in the rest of the diagonal array of *S_th_*; further, the larger the number of higher threshold values included, the larger the number of lower singular values that were excluded. At this stage, this *S_th_* matrix is considered as being in the exclusion state of lower singular values, i.e., noise component exclusion state. Using this *S_th_*—noise component excluded matrix, a denoised 4D dataset was reconstructed as follows:

$$A_{denoised}=US_{th}V^{\prime }$$

Denoised 4D CTP datasets with thresholds of 5%, 10%, 15%, 20%, 25%, 30%, 35%, and 40% were reconstructed using the SVD tool.

### 2.5. Quantitative Image Analysis

A quantitative image quality assessment of the original image and denoised images was conducted. A MATLAB software application was used to calculate the signal-to-noise ratio (SNR) and contrast-to-noise ratio (CNR) [27]. An axial image at the basal ganglia level in the enhanced phase was selected for the assessment.

To assess the SNRs at the basal ganglia level, the SNRs of a 200 × 200 region of interest were calculated using the following standard equation:

$$SNR=\frac{Mean\;\left(Region\;of\;Interest\right)}{SD\;\left(Region\;of\;Interest\right)}.$$

where, *Mean* (*Region of Interest*) denotes the average value of pixel intensities within the region of interest (ROI), and *SD* (*Region of Interest*) denotes the standard deviation of pixel intensities within the ROI.

To assess the CNRs at the basal ganglia level, the white matter (WM) and gray matter (GM) of the original and denoised images were assessed using a MATLAB tool. The locations of the WM and GM were selected in the left frontal lobe. The CNRs at the basal ganglia level were calculated using the following standard equation:

$$CNR=\frac{Mean\left(GM\right)-Mean\left(WM\right)}{\sqrt{SD{\left(GM\right)}^{2}-SD{\left(WM\right)}^{2}}},$$

### 2.6. Statistical Analysis

The statistical analysis was performed using SPSS 25.0 (IBM Corp, Armonk, NY, USA). A paired *t*-test was performed to compare variations in the quantitative image quality scores between the original image and each denoised image. Further, *p* values marked with * indicate statistical significance.

## 3. Results

### 3.1. SVD-Based Noise Removal

**Figure 3:**
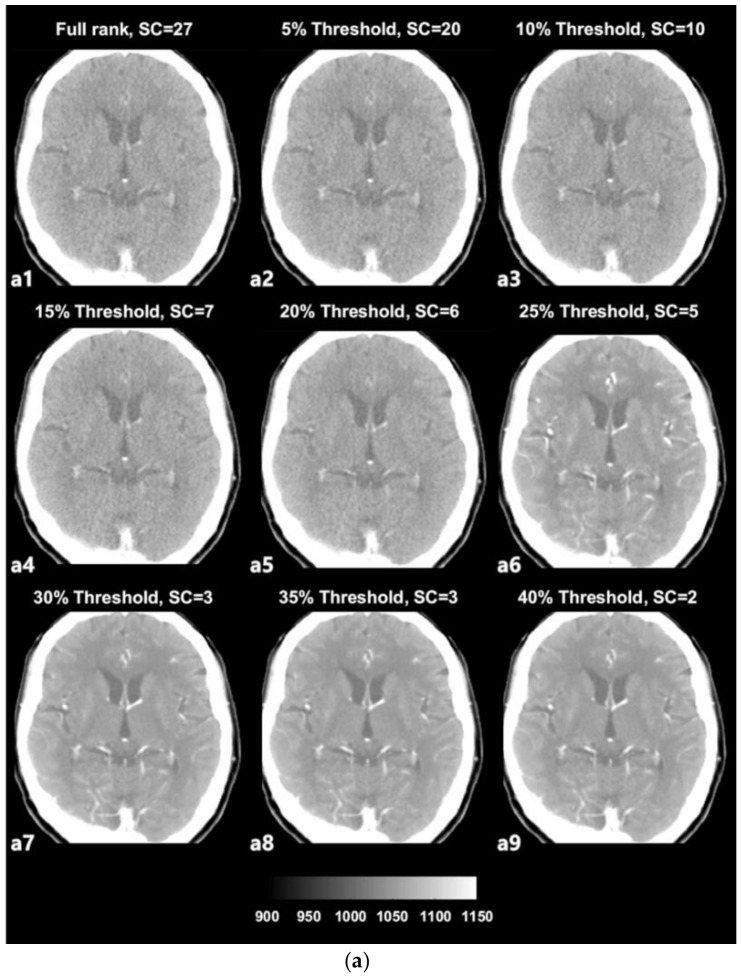

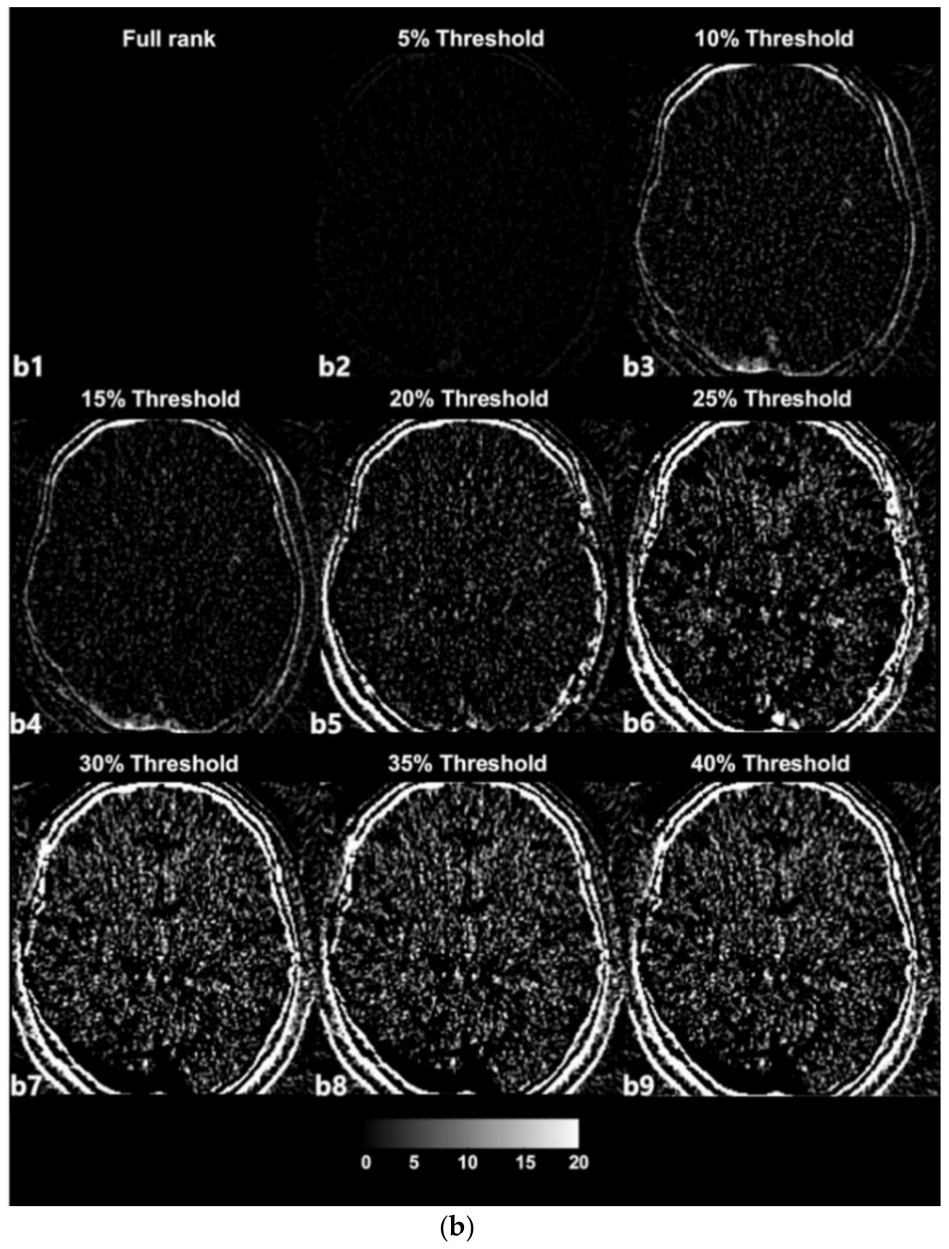
(**a**) Example of denoised images based on thresholding values; a1—original image, a2–a9—denoised images. (**b**) Example of separated noise components in accordance with thresholding; b1—no noise component was separated from the original image, b2–b9—noise components separated from the original image corresponding to Figure 3.

The SVD-based denoising technique was applied to 20 CTP datasets. Each dataset was reconstructed with singular values that were higher than the 5%, 10%, 15%, 20%, 25%, 30%, 35%, and 40% threshold values. Figure 3a depicts an example of denoised images corresponding to increasing thresholds. Figure 3b presents images with noise that were reconstructed with singular values lower than each threshold value and that were separated from the original full-rank image. In an example dataset, a full rank image (original noised Image) includes 27 singular value (Signal Component, SC) components. As shown in Figure 3a,b, the removal of larger amounts of Gaussian noise on an image is observed when the image is reconstructed with a smaller number of SCs (i.e., a larger number of low singular values were excluded).

### 3.2. Comparison with High Radiation Dose Image

**Figure 4:**
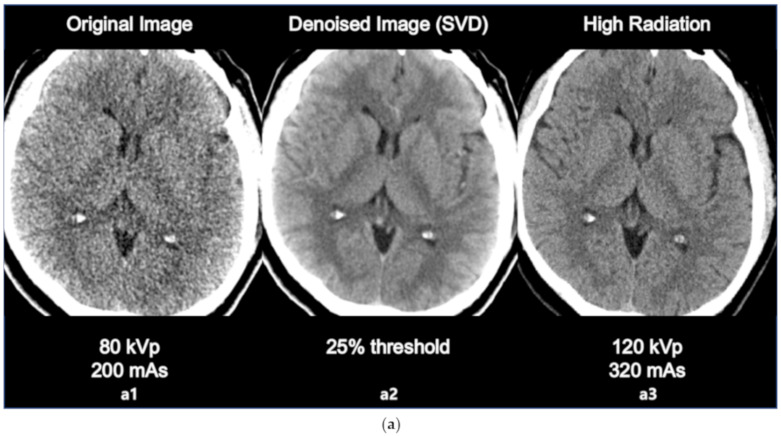

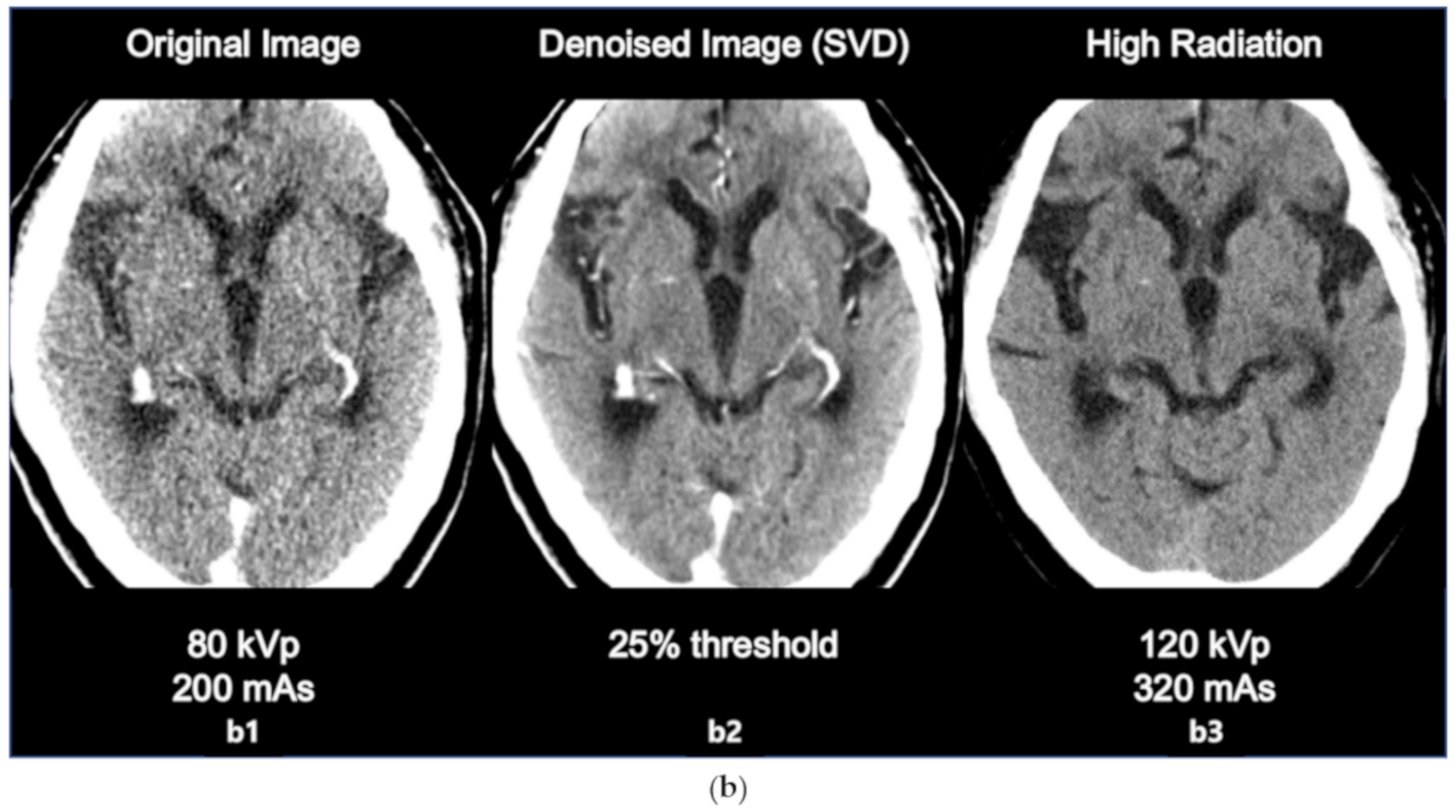
(**a**) Comparison between the original image (80 kVp, 200 mAs, a1 the denoised image using SVD (25% threshold, a2, and the high dose image (120 kVp, 320 mAs, a3). (**b**) Comparison between the original image (80 kVp, 200 mAs, b1), the denoised image using SVD (25% threshold, b2), and the high dose image (120 kVp, 320 mAs, b3).

The comparison between original image, denoised image, and high radiation dose image is as in the following Figure 4a,b. The sharpness of ventricle and parenchyma margin, the distinctness of basal ganglia and parenchyma in the denoised image, and the differentiation of gray matter and white matter in the denoised image was clearly described close to original high dose image.

### 3.3. Quantitative Analysis

**Figure 5:**
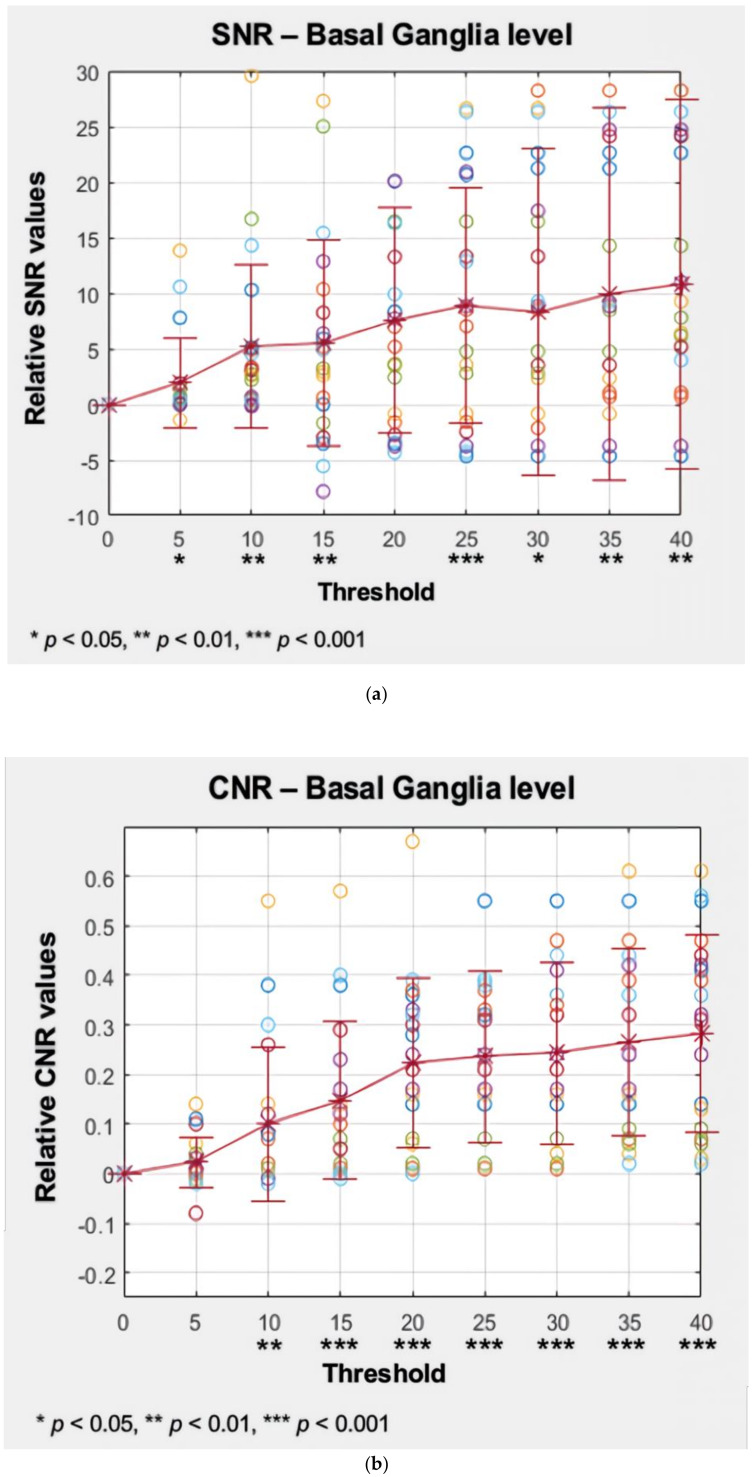
(**a**) Mean and standard deviation (SD) of the signal-to-noise ratio (SNR) at the basal ganglia level corresponding to the threshold values. (**b**) Mean and standard deviation (SD) of the contrast-to-noise ratio (CNR) at the basal ganglia level corresponding to the threshold values.

The separation of noise components from the original CTP data resulted in a significant enhancement of the SNR (Figure 5a, Table 1a) and CNR (Figure 5b, Table 1b) at the basal ganglia level. In Table 1a,b, t means the size of difference between the SNRs of each image denoised image group and the SNRs of the original image group (OG). 

In Figure 5a,b, the ‘*’ marker denotes the mean SNR and CNR value of the each image group (0; original image group with full-rank, 5–40%; and 5–40% of threshold group, respectively). The mean values were normalized considering the original image group as the zero point as shown in Table 2a,b. Each of the mean values are connected with the horizontal line, which helps to understand the tendency of increase. The vertical line overlapped on each mean value denotes the standard deviation of SNRs in each group. The point marker along the standard deviation line denotes the SNR value from single images in each group.

Accordingly, the results revealed that an increase in the SNR and CNR is associated with the number of excluded noise components, i.e., an increase in the threshold percentage setting. As a result, compared with the denoised images, the noise pollution in the original image was compensated by excluding the noise components.

## 4. Discussion

In the present study, the novel SVD-based denoising technique has been practically demonstrated on 4D CTP images of the brain. Previously, our research team reported that the SVD-based noise removal technique can be utilized for 4D CT images with only one CTP dataset [26]. Based on this study, statistical significance was demonstrated in enhancing image quality using 4D CTP datasets. We believe that this technique can be considered as a novel denoising method for 4D CTP images without the need for increasing the radiation dose. Based on our results, the use of the developed SVD tool resulted in the successful reduction of noise pollution on axial CTP images of the brain (Figure 3a). Additionally, we performed a comparison of quantitative image quality estimation results among various rank values (Figure 5a,b).

The term ‘rank’ refers to the number of singular values added to reconstruct a single image [28,29]. The number of singular values in a rank was determined in relation to the threshold value. We selected the 2nd component of the diagonal *S* matrix as the threshold value on an experiential basis; there is no golden rule to guide the selection of the threshold value because the boundaries of the signal and noise components are still vague and unclear (Figure 2). Thresholding was the process used to approximately classify the singular values in terms of signal components and noise components. The singular values that were lower than the set threshold percentage value (2nd component) were assumed to be singular value noise components in every condition and were subsequently reconstructed into a noise-containing image (Figure 3b). In contrast, the singular values that were higher than the set threshold percentage value (2nd component) were assumed to be singular signal components in every condition and were subsequently reconstructed into a denoised image (Figure 3a).

In the used dataset, the full-rank image (original image, a1) included 27 SCs (Figure 3a), i.e., the 1st to the 27th components, which were in decreasing order of magnitude and were not yet classified. In the case of Figure 3a from a2 to a9, with increasing threshold percentages, larger numbers of components were classified as noise components, resulting in denoised images. For example, in the case of (Figure 3a, a2), the 1st to the 20th singular values were included to reconstruct the denoised image labeled as the 5% threshold image, and the 21st to the 27th singular values that were lower than 5% of the 2nd component were separated from the full-rank image and used to reconstruct the separated noise-containing image (Figure 3b, a9). The image a9 that is labeled as 40% of the threshold image was reconstructed including only the 1st and 2nd singular values by separating the largest number of SCs (25 SCs) from the full-rank image. Nonetheless, the image still appears to exhibit sufficiently high image quality. Regarding image a9, the 1st and 2nd singular values are predominantly depicted as providing diagnostic information. However, it is difficult to confirm that the image at the 40% threshold is the best one, as it simply involves the separation of a larger number of noise-related patterns; reducing the noise on a medical image is inevitably a trade-off for acquiring diagnostic information.

The results of the SNR and CNR estimations are presented in Figure 5a,b. In Figure 5a, the mean SNRs at the basal ganglia level exhibited a significant increasing tendency except for the 15% and 30% threshold values. The largest improvement involving a significant 16% increase (*t* = 2.908, *p* < 0.005) was observed at the 40% threshold setting. Similarly, in Figure 5b, the mean CNRs at the basal ganglia level maintained an increasing tendency for every threshold setting. The largest improvement involving a significant 43% increase (*t* = 6.327, *p* < 0.001) was also observed at the 40% threshold setting. However, image a6 that is labeled as 25% of the threshold value resulted in the best statistical significance per the results of the paired *t* test (*t* = 3.764, *p* < 0.001), resulting in an adequate 14% increase in the SNR. Considering the example dataset presented in Figure 3a, even though the 40% setting resulted in the highest SNR, the 25% setting appeared to generate more diagnostic information associated with the vessels. The 25% setting also resulted in good CNR estimation results with a statistically significant 37% increase (*t* = 6.080, *p* < 0.001). Based on these findings, we considered that the 25% setting could potentially be the optimal setting for 4D axial CTP images at the basal ganglia level in terms of compromising between noise reduction and preserving diagnostic information. In addition, we would like to highlight that we were able to achieve a considerable improvement in quantitative image quality in the denoised images without having to increase the radiation dose. Figure 4a,b shows the comparison of the original image with the lower dose image (80 kVp, 200 mAs, a1), the denoised image (80 kVp, 200 mAs, 25% of threshold, a2), and the higher dose image (120 kVp, 320 mAs, a3). Regarding Figure 4a,b, we would like to believe that we were able to improve the diagnostic value of low dose (Figure 4a,b, a2, b2) image similar to the ones scanned with high dose (Figure 4a,b, a3, b3) by using the proposed SVD-denoising technique. In other words, there is potential to preserve sufficient image quality even in scans involving lower tube currents. Lowering the tube current and tube voltage is the simplest way to reduce the radiation dose involved in a CT scan. Based on the fact that the amount of noise on an image is inversely proportional to the square root of the radiation dose, reducing tube current and tube voltage can inevitably lead to the degradation of image quality, thereby generating a larger amount of noise on the image [30,31]. Considering this, additional studies to investigate the qualitative and quantitative comparison between the denoised lower dose image and higher dose image on CTP may be meaningful. Additionally, further study with artificial biological objects using 3D bioprinting will be required for more accurate evaluation.

The first limitation of this study is that the SVD-based technique was not applied to perfusion maps. Since the accuracy of a perfusion map is essential to detect abnormal perfusion in order to diagnose conditions, such as stroke, a follow up study needs to be conducted to investigate the effectiveness of the SVD-tool in decomposing CT perfusion maps. The second limitation of this study is the small sample size. A larger sized sample is required to further confirm our findings. Finally, we only estimated quantitative image quality; therefore, the diagnostic accuracy needs to be assessed in comparison to that of non-SVD images.

## 5. Conclusions

Our results demonstrate that the SVD-based denoising technique for 4D CTP images resulted in a considerable improvement in the quantitative image quality, SNR, and CNR, thereby implying that an increase in the radiation dose would not be necessary. Additionally, based on the results of the comparison of various images with different ranks, we consider that 25% of the 2nd component is the optimal thresholding value for axial images at the basal ganglia level in 4D CTP datasets. Further, the present study highlights the possibility that the SVD-based tool has the potential to improve the quality of images that are captured using lower tube current settings. Future studies need to perform the following: (1) use a larger sample size to support the results, (2) investigate the application of the SVD tool in decomposing CT perfusion maps, (3) investigate the effectiveness of the SVD tool on images captured with lower tube current settings, (4) evaluate the diagnostic accuracy of the denoised images, and (5) determine how an optimal threshold level can be identified in order to develop a golden standard.

## Figures and Tables

**Table 1:** (**a**) Variance from the original image and the signal-to-noise ratio at the basal ganglia level. (**b**) Variance from the original image and the contrast-to-noise ratio at the basal ganglia level.

	OG	5%	10%	15%	20%	25%	30%	35%	40%
(**a**)									
***Mean***	64.78	66.70	69.99	70.26	72.37	73.67	73.06	74.77	75.59
***SD***	20.12	21.39	21.31	23.09	23.60	23.85	25.41	25.15	25.12
***t***	0	−2.148	−3.145	−2.650	−3.344	−3.764	−2.514	−2.664	−2.908
***p***	0	* 0.022	** 0.003	** 0.008	0.002	*** <0.001	* 0.011	** 0.008	** 0.005
(**b**)									
***Mean***	0.65	0.67	0.75	0.80	0.87	0.89	0.89	0.92	0.93
***SD***	0.53	0.52	0.56	0.54	0.55	0.56	0.56	0.57	0.57
***t***	0	−1.892	−2.835	−4.146	−5.855	−6.080	−5.927	−6.304	−6.327
***p***	0	0.037	** 0.005	*** <0.001	*** <0.001	*** <0.001	*** <0.001	*** <0.001	*** <0.001

* t: the size of difference between the SNRs of each image denoised image and the SNRs original image, **: the difference between both images group are very significant, ***: the difference between both image group are highly significant.

**Table 2:** (**a**) Variance for the signal-to-noise ratio of the basal ganglia level with subtraction of OG. (**b**) Variance for the contrast-to -noise ratio of the basal ganglia level with subtraction of OG.

	OG	5%	10%	15%	20%	25%	30%	35%	40%
(**a**)									
Subject#1	0	−0.04	0.01	0.01	20.05	20.67	21.26	21.24	24.24
Subject#2	0	0.53	4.95	10.35	6.97	7.02	28.24	28.24	28.24
Subject#3	0	0.05	0.27	2.92	3.49	3.57	2.34	2.34	6.42
Subject#4	0	0.72	0.67	12.87	20.11	20.92	17.41	24.72	24.72
Subject#5	0	0.02	2.73	3.25	3.64	4.76	4.76	4.76	6.15
Subject#6	0	0.48	4.55	−5.56	−4.34	−4.29	−35.36	−35.36	−35.36
Subject#7	0	0.67	3.11	−2.98	−2.74	−2.47	3.54	3.54	5.19
Subject#8	0	7.80	10.29	−3.51	−3.51	−4.65	−4.65	−4.65	−4.65
Subject#9	0	0.12	3.34	5.18	5.18	8.52	8.52	1.05	1.05
Subject#10	0	−1.39	29.56	27.30	34.51	26.63	26.63	43.70	43.70
Subject#11	0	0.37	−0.16	−7.83	−3.75	−3.75	−3.75	−3.75	−3.75
Subject#12	0	0.63	16.69	25.02	16.48	16.47	16.47	8.49	7.81
Subject#13	0	10.58	14.30	15.43	16.29	26.32	26.32	26.32	26.32
Subject#14	0	1.91	3.08	8.27	13.28	13.31	13.31	24.14	24.14
Subject#15	0	0.05	5.14	5.90	8.36	22.63	22.63	22.63	22.63
Subject#16	0	0.04	0.47	0.61	−1.61	−1.57	−2.14	0.69	0.69
Subject#17	0	13.84	2.60	2.60	−0.86	−0.84	−0.84	−0.84	9.27
Subject#18	0	0.02	0.01	6.38	7.74	8.84	8.84	8.84	10.98
Subject#19	0	1.45	2.19	−1.69	2.42	2.81	2.81	14.26	14.26
Subject#20	0	0.40	0.37	4.93	9.91	12.89	9.26	9.26	3.97
***Mean***		1.91	5.21	5.47	7.58	8.89	8.28	9.98	10.80
***SD***		3.88	7.22	9.00	9.88	10.30	14.35	16.33	16.19
***t***		−2.148	−3.145	−2.650	−3.344	−3.764	−2.514	−2.664	−2.908
***p***		* 0.022	* 0.003	* 0.008	0.002	* 0.001	* 0.011	* 0.008	* 0.005
(**b**)									
Subject#1	0	0.01	0.01	0.01	0.28	0.32	0.32	0.32	0.41
Subject#2	0	0.01	0.07	0.23	0.33	0.33	0.47	0.47	0.47
Subject#3	0	0.00	0.01	0.05	0.06	0.07	0.04	0.04	0.03
Subject#4	0	−0.01	−0.01	0.12	0.33	0.38	0.41	0.42	0.42
Subject#5	0	0.00	0.01	0.02	0.02	0.02	0.02	0.02	0.02
Subject#6	0	0.06	0.30	0.40	0.39	0.39	0.36	0.36	0.36
Subject#7	0	0.10	0.26	0.29	0.30	0.31	0.32	0.32	0.31
Subject#8	0	0.11	0.08	0.14	0.14	0.14	0.14	0.14	0.14
Subject#9	0	0.00	0.02	0.01	0.01	0.01	0.01	0.07	0.07
Subject#10	0	0.06	0.55	0.57	0.67	0.55	0.55	0.61	0.61
Subject#11	0	0.03	0.12	0.23	0.24	0.24	0.24	0.24	0.24
Subject#12	0	−0.01	0.01	0.07	0.07	0.07	0.07	0.09	0.09
Subject#13	0	0.01	0.01	0.00	0.00	0.02	0.02	0.02	0.02
Subject#14	0	−0.08	0.01	0.05	0.21	0.21	0.21	0.44	0.44
Subject#15	0	0.00	0.38	0.38	0.36	0.55	0.55	0.55	0.55
Subject#16	0	0.00	0.02	0.10	0.37	0.37	0.34	0.39	0.39
Subject#17	0	0.14	0.14	0.14	0.16	0.16	0.16	0.16	0.13
Subject#18	0	0.01	0.01	0.17	0.17	0.17	0.17	0.17	0.32
Subject#19	0	0.04	0.01	−0.01	0.02	0.02	0.02	0.06	0.06
Subject#20	0	−0.02	−0.02	−0.01	0.32	0.38	0.44	0.44	0.56
***Mean***		0.02	0.10	0.15	0.22	0.23	0.24	0.27	0.28
***SD***		0.05	0.15	0.16	0.17	0.17	0.18	0.18	0.19
***t***		−1.892	−2.835	−4.146	−5.855	−6.080	−5.927	−6.304	−6.327
***p***		* 0.037	* 0.005	* <0.001	* <0.001	* <0.001	* <0.001	* <0.001	* <0.001

*: the difference between both images group are significant.

## Abbreviations

SC	singular component
